# The advantages and limitations of guideline adaptation frameworks

**DOI:** 10.1186/s13012-018-0763-4

**Published:** 2018-05-29

**Authors:** Zhicheng Wang, Susan L. Norris, Lisa Bero

**Affiliations:** 1 Faculty of Medicine and Health, The University of Sydney, Sydney, New South Wales Australia; 2 World Health Organization, Geneva, Switzerland; 3 Charles Perkins Centre, The University of Sydney, D17, The Hub, 6th floor, Sydney, New South Wales Australia

**Keywords:** Guidelines, Adaptation, Global health, Adaptation frameworks

## Abstract

**Background:**

The implementation of evidence-based guidelines can improve clinical and public health outcomes by helping health professionals practice in the most effective manner, as well as assisting policy-makers in designing optimal programs. Adaptation of a guideline to suit the context in which it is intended to be applied can be a key step in the implementation process. Without taking the local context into account, certain interventions recommended in evidence-based guidelines may be infeasible under local conditions. Guideline adaptation frameworks provide a systematic way of approaching adaptation, and their use may increase transparency, methodological rigor, and the quality of the adapted guideline.

This paper presents a number of adaptation frameworks that are currently available. We aim to compare the advantages and limitations of their processes, methods, and resource implications. These insights into adaptation frameworks can inform the future development of guidelines and systematic methods to optimize their adaptation.

**Analysis:**

Recent adaptation frameworks show an evolution from adapting entire existing guidelines, to adapting specific recommendations extracted from an existing guideline, to constructing evidence tables for each recommendation that needs to be adapted. This is a move towards more recommendation-focused, context-specific processes and considerations. There are still many gaps in knowledge about guideline adaptation. Most of the frameworks reviewed lack any evaluation of the adaptation process and outcomes, including user satisfaction and resources expended. The validity, usability, and health impact of guidelines developed via an adaptation process have not been studied. Lastly, adaptation frameworks have not been evaluated for use in low-income countries.

**Conclusion:**

Despite the limitations in frameworks, a more systematic approach to adaptation based on a framework is valuable, as it helps to ensure that the recommendations stay true to the evidence while taking local needs into account. The utilization of frameworks in the guideline implementation process can be optimized by increasing the understanding and upfront estimation of resource and time needed, capacity building in adaptation methods, and increasing the adaptability of the source recommendation document.

**Electronic supplementary material:**

The online version of this article (10.1186/s13012-018-0763-4) contains supplementary material, which is available to authorized users.

## Background

Guidelines can be defined as “any document containing recommendations for clinical practice or public health policy. A recommendation tells the intended end-user of the guideline what he or she can or should do in specific situations to achieve the best health outcomes possible, individually or collectively” [1]. Guidelines are developed by a range of organizations including charities endorsed by local professional societies (e.g., The Heart Foundation endorsed by the Royal Australian College of General Practitioners (RACGP)), national health research institutes (e.g., US National Institutes of Health (NIH), the UK National Institute for Health and Care Excellence (NICE), and Australian National Health and Medical Research Council (NHMRC)), and international health organizations (e.g., the World Health Organization (WHO)). In order to be trustworthy, all guidelines, both clinical and public health, should be evidence based and should be developed using clear, explicit processes to minimize bias and optimize transparency [2].

### Guideline implementation

The implementation of evidence-based guidelines can improve clinical and public health outcomes by helping health professionals practice in the most effective manner [3, 4], as well as assisting policy-makers in designing optimal programs. The development of guidelines without adequate consideration of implementation may hinder the target audiences’ adherence to the guidelines [5]. Without proper implementation, the financial and human resources expended in the development of guidelines are wasted.

The implementation of guidelines in a context that is different from where they were developed is particularly challenging. In addition, recommendations in public health guidelines are often more complex to implement than clinical guidelines and usually target health systems or multi-sector government institutions instead of individual clinical decisions. For example, WHO develops guidelines for a global audience; each guideline or recommendation in each guideline then needs to be considered for implementation at the country or sub-national level (e.g., within a health system). Other examples include implementation of national guidelines to the local (e.g., state or provincial) context [6], international guidelines to a local hospital [7], European guidelines to individual countries [8], and international guidelines to regions [9].

There are a number of systematic reviews on the effectiveness of various implementation strategies for recommendations in guidelines [10–12]. Most indicate that active techniques are the most effective. However, many guidelines do not include detailed descriptions of how the guidelines should be implemented [13].

### Guideline adaptation

Adaptation is a key step in the implementation process [13]. Guidelines International Network (G-I-N) defines guideline adaptation as “the systematic approach to the modification of a guideline(s) produced in one cultural and organisational setting for application in a different context”. Guideline adaptation is usually initiated by end-users at the local level (e.g., by local governments, hospitals, and/or individual clinicians) and not by international (e.g., WHO) or national (e.g., NHMRC) guideline developers. Adaptation is an alternative to de novo guideline development such as customizing an existing guideline to the local context [14] which could be a specific health setting, country, or an emergency situation. In order to achieve effective adaptation, guideline adaptors should take into account a number of important aspects of the local context such as resource capabilities (both human and material), disease prevalence, and the values and preferences of community members.

If the local context is not taken into account, interventions recommended in existing high-quality guidelines may be impossible to implement. For example, recommending widespread use of information and communications technologies without adequate knowledge of their use in the local health system may be more of a burden than a boon to the health system [15]. Adapting the guidelines and local capacity building in understanding and applying the recommended interventions are vital for their successful uptake. It is not only the recommendations within the guidelines that may need to be adapted to suit the local context, but also different implementation strategies may be required for guidelines in different contexts.

Developing guidelines de novo requires substantial time and resources—both methodological expertise and fiscal capacity. When a high-quality guideline is available which addresses the local need, it may be more practical to adapt this guideline (or selected recommendations therein) for local use [16]. For example, until 2012, New Zealand had a high-quality internationally respected guideline development program through the New Zealand Guidelines Group [17]. This group went into voluntary liquidation in mid-2012 [17]. After this, the New Zealand Ministry of Health provided funding to a new guideline organization: The Best Practice Advocacy Centre New Zealand (bpac^NZ^) to adapt NICE clinical guidelines for use in New Zealand based on the ADAPTE approach [18].

From here on, we will refer to original and established source materials (e.g., WHO guidelines) as “source guidelines” or “source materials,” while the new and modified guidelines/recommendations produced by the adaptation process will be referred to as “adapted guidelines” or “adapted recommendations.”

When a clinical practice or public health guideline is needed in a specific context, recommendations can be constructed using one of four possible approaches:

1. Adopt recommendations from existing evidence-based source guidelines without modification;
2. Adapt recommendations from existing guidelines to the new context;
3. Develop recommendations de novo based on existing reviews of evidence (from source guidelines or systematic reviews) [19]; and
4. Develop recommendations de novo based on new evidence syntheses.

Adapted guidelines can contain recommendations from a mixture of these approaches. Additional file 1 summarizes factors that may influence a local group to choose one of these approaches over another.

### Forms of adaptation

Guideline adaptation occurs via either informal or formal processes.

#### Informal adaptation

Informal guideline adaptation occurs without using an established framework [7]. For example, when a hospital in Lebanon considered adapting a guideline on low back pain [7], no formal adaptation framework was used. The hospital guideline adapters simply identified international guidelines in the literature, compared them according to the AGREE instrument [20], and implemented the “best” one after translating it into the local language [7].

Informal adaptation can also be done on an individual provider or patient level [21]. Doctors in Sudan were noted to adapt international guidelines on an ad hoc basis, in order to suit the patient and the health care system in their country. One of the doctors interviewed in this study said “I cannot prescribe the new drug (X) which is not found in Sudan. We stick to guidelines but with a modified picture” [21]. The high frequency of testing suggested by international guidelines may also be impractical in low-resource settings, as for example, some patients may have to travel long distances for the tests [21].

Such ad hoc adaptations, although practical in some situations, can pose a risk if the intervention that is implemented is outside of the scope of the original evidence-based recommendation.

#### Formal adaptation

This occurs when adaptation of a guideline is performed using a guideline adaptation group and an established framework [22]. Table 1 lists possible steps in an adaptation framework.

**Table 1:** Possible steps in an adaptation framework

1) Form an organizing committee.	
2) Choose a guideline topic.	
3) Identify resources and skills required for the process.	
4) Write an adaptation plan and form a guideline adaptation group.	
5) Determine the health questions.	
6) Search for relevant guidelines and related documents.	
7) Formally screen and review (i.e., assess currency, content, quality, consistency between sources and acceptability/applicability of the recommendations) selected guidelines.	
8) Decide which guideline or recommendations to adapt, taking into account the quality of the source material, local conditions, and practicality of the guideline/recommendations/intervention.	
9) Perform external review of the adapted guideline (by target audience, endorsement bodies, and source guideline developers).	
10) Schedule evidence reviews and updates of the adapted guideline.	

This framework summary is based on ADAPTE [16]

Formal adaptation frameworks provide a systematic way of approaching adaptation. These frameworks are created to increase methodological rigor and quality of the adapted guideline [23]. Due to the complexity of applying formal frameworks, this type of adaptation is always done collectively. Formal frameworks, in contrast to informal adaptation methods, can enable evaluation of the evidence supporting the recommendations in adapted guidelines. A recent review has identified some of the frameworks for guideline adaptation [24].

## Aim

We aim to understand advantages and limitations of existing frameworks and identify knowledge gaps in the process of guideline adaptation through an analysis of recent adaptation frameworks. This in turn will help to identify optimal characteristics of frameworks to inform guideline development, implementation, and uptake.

## Analysis

### Description and critique of adaptation frameworks

The analysis drew from literature published over the past 15 years (1 January 2002 to 1 March 2017) as this area of research is relatively new. Very few studies on guideline adaptation were published prior to 2002. MEDLINE, Embase, and CINAHL databases were systematically searched for published accounts of formal adaptation frameworks. The search strategy can be found in the Additional file 1.

The results were limited by language (English) and publication type (clinical trial, journal article, meta-analysis, randomized controlled trial, research, review, systematic review, multicentre study, or observational study) and population (Humans NOT animals). The titles of the results were screened for relevance.

As little work has been done to review this area, the results were screened in a scoping review style without limits on types of articles we would include or a priori protocols of analytical categories of data extraction (specific features of adaptation frameworks) [25]. The categories and the inclusion and exclusion criteria were refined as the data were collected. We identified eight different frameworks, many of which were developed concurrently or build on each other.

#### Timeline of frameworks

**Fig. 1:**
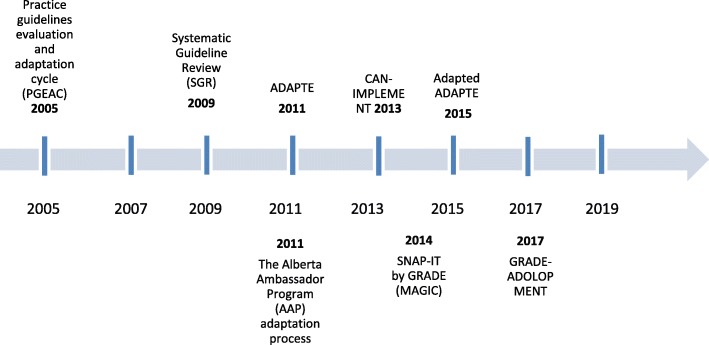
Timeline for publication of adaptation frameworks. A brief timeline of the publication dates of the frameworks examined in the paper. Some later frameworks built on the works of previous ones. Note that certain frameworks may have been available before the publication date

The timeline of framework development is illustrated in Fig. 1. The authors overlap in some of the frameworks (Harrison, M. B. and Graham, I. D. worked on PGEAC, ADAPTE, and CAN-IMPLEMNT), which may explain some of the similarities among the early frameworks.

#### Similarities and differences in processes of adaptation suggested by the frameworks

As shown in Table 2, there are similarities and differences in the adaptation processes suggested by the different frameworks.

**Table 2:** Processes of adaptation suggested by published frameworks

Framework (year published) Author(s) (framework development group)	Committee structure	Methods and process summary	Updating of the adapted guideline	How adapted recommendations were constructed (e.g., consultation, consensus, EtD tables)	External peer review	Presentation and dissemination of the adapted guideline
Practice guideline evaluation and adaptation cycle (2005) [36] Graham, I. D. Harrison, M. B.	A single local interdisciplinary20 guideline evaluation group comprising key stakeholders	• Identify a clinical area to promote best practice, • Search and evaluate existing guidelines, • Adopt or adapt the guideline for local use	Yes	Consensus in the guideline evaluation group	Yes, by local practitioners, other stakeholders, and organizational policy-makers for review and comment	Unclear, likely hardcopy documents
Systematic guideline review (2009) [37] Muth, C. et al. (“Kompetenznetz Herzinsuffizienz” and the German Society for General Practice and Family Medicine (DEGAM))	Most steps conducted by the 5 authors	• Use multiple sources to search for guidelines • Assess quality of the guidelines • Collate recommendations from different guidelines into “evidence tables of a standardized format which included recommendation(s), evidence level(s), grading, critical appraisal of evidence, and cited sources”	No	Consensus by the authors	Yes, a multi-professional, interdisciplinary formal consensus process that included a patients’ representative and a pilot testing phase	Hardcopy documents
ADAPTE (2011) [16] ADAPTE Collaboration (including Graham, I. D. Harrison, M. B.)	Dual committee structure consisting of the organizing committee and panel of guideline developers (usually content experts)	• Search for source guidelines • Assess source guidelines • Adapt source guideline	Yes	Consensus by the panel	Yes, by target users, consulted with relevant endorsement bodies and the developers of source guidelines	Hardcopy documents
The Alberta Ambassador Program (AAP) adaptation process (2011) [26] Harstall, C. et al. (The Alberta Ambassador Program)	Up to 6 committees with distinct responsibilities in the adaptation process	• Formulate the question from knowledge gaps in the adaptation context • Literature search to identify relevant source guidelines. • Assess source guidelines • Adapt guideline written via monthly videoconferences of the Guideline Development Group	Yes “living” guideline that will be updated every 2 years	Consensus by the guideline development group	Yes, by clinical experts, methodologists, and potential guideline users who were not involved in its development	Targeted to local implementation facilitators. Including internet access to the guidelines
CAN-IMPLEMENT (2013) [30, 38] Harrison, M. B. Graham, I. D. et al. (The Canadian Partnership Against Cancer)	2 or more committees including a steering committee and working panel(s)	• Similar steps to ADAPT • Some steps of the adaptation process done simultaneously by different sub-committees of the guideline development group. • A stronger focus on the implementation of guidelines after their adaptation	Yes	Consensus by the panel	Yes, by each stakeholder group affected by the recommendations	Adaptation only the first phase of the CAN-IMPLEMENT process. Phase 2 is development of training programs and interventions to implement new guideline. Phase 3 involves evaluation of the process and outcomes
SNAP-IT by GRADE (2014) [28] Kristiansen, A. et al. (Canadian McMaster University GRADE group partnership with Norwegian Ministry of health)	Editorial committee, individual chapter editors	• Select one well established guideline which was deemed to be current, of high quality, and used GRADE (23) • Choose recommendations within this guideline that they deem relevant in the adaption context to adopt/adapt	Dynamically update the recommendations at least every 3 months	One content expert and one methods expert on the editorial committee reviewed each chapter of the guideline to choose which recommendations to adopt and/adapt. The panel consulted with editors of the source guideline on content issues and when modifications were made.	Yes, by all relevant medical specialty organizations, local ministry of health and the source guideline development organization	Published in newly developed web authoring and publication platform (MAGIC), including offline access on smartphones and tablets
Adapted ADAPTE (2015) [32] Amer, Y. S. Elzalabany, M. M. Omar, T. I. Ibrahim, A. G. Dowidar, N. L.	Dual committee structure consisting of the organizing committee and panel	Framework based on the work of ADAPTE collaboration and CAN-IMPLEMENT with modifications to increase the timeliness and clarity of the adaptation process	Yes	Consensus by the panel	Yes, same as ADAPTE	Hardcopy documents. The framework include some implementation tools which include professional and organizational interventions, monitoring and evaluation, and an action plan for dissemination
GRADE-ADOLOPMENT (2017) [19] Schunemann, H. J et al. (Canadian McMaster University GRADE group partnership with Saudi Arabian Ministry of health)	Methodologist group from McMaster university. Guideline panels made up of local expert members from multidisciplinary backgrounds, including some patient representatives	• Local authorities choose the key clinical questions; • Identify specific recommendations that address those questions. • Choose source guidelines based on the GRADE approach and constructing EtD tables • Revise and update these tables are to match the local context.	N/A This framework stops at the decision to either adopt, adapt the source recommendation/evidence, or start de novo development of a new guideline.	Evidence to decision (EtD) tables	N/A This framework stops at the decision to adopt, adapt the source recommendation/evidence or start de novo development of a new guideline.	Unclear, in the case described the adapted guidelines were made for the Kingdom of Saudi Arabia and dissemination was the responsibility of the local government

*Abbreviations: EtD* evidence-to-decision, *GRADE* The Grading of Recommendations Assessment, Development and Evaluation, *N/A* not applicable

The frameworks differ in the structure of the committees that conduct the adaptation, with a number suggesting two committees (organizers and guideline developers) (e.g., ADAPTE [16]), while later frameworks tend to have more complex structures (e.g., AAP [26]). The steps of the adaptation process also differed greatly, particularly with respect to how adaptation panels were selected and how they evaluated source materials; these differences will be explored further in the following section.

The frameworks also differed in how the adapted recommendations were constructed, although consensus by the panel was the most common process. The requirements of external review and plans for updating the adapted guideline were almost universal in the eight frameworks. The frameworks usually suggested disseminating hardcopies of the adapted guidelines.

#### Processes for selecting and evaluating source materials

Processes for identifying and evaluating source materials for adaptation differed significantly across the frameworks (see Table 3). One point of major divergence is in the processes used to search for and evaluate the source material used in the adaptation process. Frameworks have evolved from a focus on identifying source guidelines for adaptation to identifying specific recommendations for adaptation. The frameworks then evolved from a focus on recommendations to examining the evidence underpinning the adapted recommendations.

**Table 3:** Processes for identification and evaluation of source material by adaptation frameworks

Framework	Define the health question	Search and screen	Evaluate guidelines	Identify recommendations	Evaluate recommendations	Identify new evidence	Evaluate evidence
Practice guidelines evaluation and adaptation cycle (PGEAC) [36]	Select a clinical question based on: • The prevalence of the condition or its associated burden • Concerns about large variations in practice or care gaps, • Costs associated with different practices	US National Guideline Clearinghouse and guideline repositories, as well as guideline developers and PubMed [36]	AGREE	N/A	If more than one guideline is being considered, a “content analysis” of the recommendations in each guideline is conducted by clinicians experienced in the content area. A table is used to compare the recommendations in each guideline and the level of evidence supporting each recommendation	N/A	N/A
Systematic guideline review (SGR) [37]	Not specified by the framework. Chronic Heart Disease was the topic already chosen for the review.	MEDLINE, The Cochrane Library, DARE, and HSTAT [37]	AGREE	For each clinical question extract data into evidence tables including: recommendations, evidence levels, grading, critical appraisal of evidence, and cited sources.	Recommendations within the guidelines are evaluated for whether they are supported by valid study results	N/A	Systematic reviews cited by the source guidelines are re-evaluated, along with clinical studies of an appropriate design when secondary publications did not provide the desired evidence
ADAPTE [16]	Topic chosen before the adaptation process. Research questions determined by the guideline committee in the patient population, intervention, professional/patients (audience of the guideline), outcomes, and healthcare setting (PIPOH) format [16]	26 guideline internet sites including the Cochrane Library, guideline repositories, government agencies and cancer clinical societies [39]	AGREE, Assess guideline currency, content and consistency	Construct recommendation matrices with a list of recommendations and their respective source guidelines to allow comparison of the recommendations	Assess acceptability (i.e., whether the recommendations should be put into practice) and applicability (i.e., whether an organization or group is able to put the recommendation into practice). Assess consistency between the evidence cited by the guidelines and the respective recommendations.	N/A	N/A
The Alberta Ambassador Program (AAP) adaptation process [26]	Knowledge gaps of the local practitioners were assessed along with a systematic review of the literature on knowledge gaps among various primary case groups	Search developed by research team in collaboration with experienced medical librarians (28)	AGREE modified by the research team	Evidence inventory tables are used by the research team to extract data from source guidelines and present all the information required for the guideline development group in 1 document. Discordant recommendations are highlighted	Not assessed, evidence cited in the source guidelines to support the recommendations is listed.	N/A	N/A
CAN-IMPLEMENT from ADAPTE [30, 38]	Topic chosen before the adaptation process. Research questions determined by the guideline committee in the Patient population, Intervention, Professional/patients (audience of the guideline), Outcomes; and Healthcare setting (PIPOH) format [30]	Guideline clearinghouses, country-specific databases, relevant specialty societies and web sites of organizations developing guidelines. MEDLINE, Google, AltaVista, and Yahoo [38]	AGREE II assess guideline currency, content, and consistency between evidence and recommendations [40]	Construct a table or “matrix” which compares similar recommendations across multiple guidelines and displays relative levels of evidence	Acceptability and Applicability of recommendations; consistency between the developers’ selected evidence, interpretation, and resulting recommendations	N/A	N/A
SNAP-IT by GRADE [28]	Guideline topic requested by the local health authorities	N/A	N/A	A designated chapter editor assessed each chapter and decided whether to adopt or adapt the recommendation.	The chapter editors then follow a predefined taxonomy to decide whether to adopt, adapt or develop a new recommendation.	N/A	If the panel decided to exclude or modify a recommendation, a more extensive reassessment of the underlying evidence is conducted.
Adapted ADAPTE [32]	Determined by the guideline committee in the Patient population, Intervention, Professional/patients, Outcomes; and Healthcare setting (PIPOH) format [32])	Seven CPG resources prioritized from the original 26-long list in ADAPTE and “DynaMed”, BMJ Best Practice and PubMed [32]	AGREE II	Construct a list of recommendations and their respective source guidelines to allow comparison of the recommendations	Assessment done when tailoring more than one guideline that includes selecting some, not all, recommendations from different source guidelines. Consistency between the evidence cited by the guidelines and the respective recommendations is assessed.	N/A	N/A
GRADE-ADOLOPMENT [19]	Guideline topic selected by the local health authorities	N/A	N/A	Take recommendations from existing guidelines that used the GRADE approach and had publicly available evidence summaries in the form of GRADE Summary of Findings (SoFs) tables or evidence profiles (EPs)	Assess each recommendation in EtD tables. The EtDs included the summary of evidence about the benefits and harms of the intervention option(s) and information about the importance of the problem (e.g., baseline risk), patients’ values and preferences, resource use, costs, feasibility, acceptability, and potential impact on health equity of recommending specific intervention options in the context and affected stakeholders	Evidence syntheses related to the existing recommendations were searched for; including systematic reviews and HTAs.	Systematic reviews are updated if the source systematic reviews are older than 3 months and the results fed into the EtD tables. The quality of the evidence was rated using GRADE

Note. AGREE II was published in 2010

*Abbreviations: AGREE* Appraisal of Guidelines for Research and Evaluation; *CPG* clinical practice guidelines; *EtD* evidence-to-decision; *GRADE* The Grading of Recommendations Assessment, Development and Evaluation; *HTA* Health Technology Assessment; *N/A* not applicable

The initial steps of the guideline adaptation process are similar among the early frameworks (PGEAC, SGR, ADAPTE, AAP, CAN-IMPLEMENT, and Adapted ADAPTE) as they all used a selection of guidelines as their source material. This process can be summarized as:

1. Define the health questions
2. Search and screen the guidelines
3. Evaluate the guidelines
4. Select the single or a set of guideline/s to adapt

These earlier frameworks use versions of the AGREE tool to evaluate the selected guideline [20, 27]. AGREE assess the following domains:

1. Scope and purpose
2. Stakeholder involvement
3. Rigor of development
4. Clarity of presentation
5. Applicability
6. Editorial independence (conflicts of interest of members of the guideline development group)

The SNAP-IT by GRADE framework differs from the others as it does not select and evaluate a range of guidelines. Instead, this framework suggests selecting a single well-known guideline, then modifying the recommendations for the local context [28]. For example, the guideline “Antithrombotic Therapy and Prevention of Thrombosis, 9th ed: American College of Chest Physicians Evidence-Based Clinical Practice Guidelines (AT9)” was chosen as it was current and the “largest CPG to rigorously apply the GRADE methodology, providing authoritative assessments of confidence in evidence and explicit rationales for the strength of its recommendations” [28]. This framework has many similarities to the GRADE-ADOLOPMENT developed years later by the same group.

Adaptation frameworks are evolving towards identifying and evaluating recommendations within a single large guideline. Increasing, the frameworks also started to make explicit the multiple paths that the adaptors can take to construct adapted recommendations. For example, in SNAP-IT by GRADE, the panelists were designated a chapter in the source guideline to evaluate and they “reviewed each recommendation in their designated chapter and formally recorded their views regarding whether the recommendation could stand as it was or whether there was a need for modification, exclusion, or development of new recommendations” [28].

GRADE-ADOLOPMENT was the first framework to make a distinction between adopting and adapting a guideline. It describes three paths: (1) adopt existing recommendations as they are, (2) adapt existing recommendations to their own context, and (3) develop recommendations de novo based on available evidence syntheses [19]. These pathways were evident in a less elaborate form in SNAP-IT by GRADE. GRADE-ADOLOPMENT goes further in that it not only searches for guidelines or recommendations in guidelines, it selects existing “highly credible guidelines and evidence syntheses, including systematic reviews and [health technology assessments] HTAs” [19]. The evidence from all these sources is used to construct GRADE evidence-to-decision (EtD) tables which include updated evidence syntheses on intervention effects, with particular attention to the local health care setting and key context-specific factors [19]. Recommendations were then formulated based on the EtD tables, via consensus or voting when necessary.

This demonstrates that frameworks have evolved from a focus on recommendations to examining the related evidence (e.g., systematic reviews and HTAs). Few frameworks described the methods used to assess whether and how a specific recommendation should be adapted.

#### Limitations of adaptation frameworks

We identified several limitations to using the various adaptation frameworks. Firstly, there is minimal guidance about the costs or time required for frameworks like ADPATE [29]. Without a clear understanding of how much time and resources adaptation frameworks actually save, guideline developers cannot be sure that a framework is worth using [30]. The frameworks are reported to be time and resource intensive [16, 28–32], despite their original purpose being to increase efficiency and reduce duplication of effort compared to de novo guideline development [16]. Each project can take from 3 years using adapted ADAPTE [32] to 18 months using ADAPTE [29].

To address the lengthy timeframe required for the ADAPTE framework, the CAN-IMPLEMENT framework involves conducting concurrent tasks by multiple, collaborative groups to reduce the duplication of effort [30]. By delegating tasks according to expertise of guideline development group members, the workload can be shared. Additionally to address the need for methodological expertise, the CAN-IMPLEMENT team suggests outsourcing and consultations with specialists (e.g., library science, evidence appraisal) when required [30].

A second limitation is that the frameworks require a level of methodological expertise which is not available to many guideline development groups [29]. Guideline developers may need a specific methods or research team separate to the guideline development group that can present evidence to the guideline development group for analysis and discussion [26, 29]. To address this challenge, the Alberta Ambassador Program implements a complex array of committees that oversee different tasks in the guideline adaptation process [31]: Steering and Advisory Committees for oversight, a guideline development group to construct the adapted guideline, and a research team to select and appraise published guidelines, prepare background documents, and assist with writing the adapted guideline [31]. This structure has proven problematic, however, with high rates of attrition of committee members and confusion among participants about their roles [31].

## Gaps in knowledge about the process of guideline adaptation

Our analysis of guideline adaptation frameworks has identified a few gaps in knowledge about the process of guideline adaptation. Firstly, the guideline adaptation frameworks examined in this study have been applied primarily in high- and upper middle-income countries and most were developed by large, experienced collaborations such as the GRADE Working Group [19, 28]. Only one framework (i.e., adapted ADAPTE [32]) has been applied in a lower middle-income setting. Thus, studies of guideline adaptation in low- and middle-income countries are needed, including exploration of the needs for, and barriers and facilitators of, guideline adaptation. Future studies can explore what pragmatic and efficient processes can be used in resource-limited settings to product valid and impactful adapted guidelines. Adaptation of a guideline in a high-income country may differ from a low-income country because low- and middle-income countries may have a more severe lack of human and fiscal resources [32]. The health systems in many low- and middle-income countries may also have practical issues that need to be addressed in the guidelines (e.g., medication/staff shortage, hospital overcrowding, inequity in care delivery) [33].

Secondly, most of the frameworks reviewed lack any formal evaluation. In the few instances where evaluations have been performed, they mostly focused on perceived usability of frameworks through self-administered surveys of the guideline developers [16], reflections from the adaptation process recorded in a “lesson-learned log” [29], and interviews with the participants in the process [30]. These self-administered evaluations are not adequate measures for the quality of the frameworks.

Thirdly, although the lack of methodological expertise in the developers was cited as a major barrier to the frameworks’ usability, there were no formal evaluations as to how having a research team with methodological expertise could have improved the particular framework [29].

Fourthly, it is unclear whether the shortcuts taken in the frameworks affect the resulting adapted guidelines. The process of adaptation is meant to expedite the process of constructing context-relevant guidelines compared to de novo development. For example, the SNAP-IT framework [28] skips the guideline search-and-select process of all previous frameworks, thus saving time and resources. By going straight to the evidence (using EtD tables) for the relevant recommendations [19], the GRADE-ADOLOPMENT framework integrates the evidence appraisal process into the formation of the adapted recommendations. The impact of these changes in the frameworks on the validity of the resultant recommendations and the advantages in terms of resources expended are unknown.

Fifthly, a common missing element in adaptation frameworks, even in the most recent ones, is that they do not advise developers how to implement the adapted guideline [19]. GRADE-ADOLOPMENT recognizes the importance of involving local stakeholders in the adaptation process [19]; however, this still leaves the local health workers and policy-makers on their own to implement adapted guidelines.

### Addressing limitations and gaps in guideline adaptation

More research in guideline adaptation and the use of frameworks in low- and middle-income countries will increase knowledge and experience in the area. Due to the unique challenges of these settings, frameworks could be a great tool for improving health outcomes or a great burden for the local health system. Health care systems of low- and lower middle-income countries generally have a shortage of specialized groups and resources for development or adaptation of guidelines [32]. This calls for greater assistance from international guideline developers (e.g., WHO) to partner with local institutions and/or governments to adapt evidence-based practice guidelines to local settings.

More independent tests need to be performed to evaluate the usability of the frameworks as well as to assess the effectiveness of the frameworks in improving guideline implementation and uptake in different settings. An example of an independent test done to evaluate a framework can be found in the NHMRC’s adaptation of physical activity guidelines using the GRADE ADOLOPMENT framework [34]. Where through their experience of adapting a guideline using the framework, the NHMRC provided suggestions to improve the GRADE ADOLOPMENT approach. With better evaluation, the quality of frameworks could be better modified and continually refined to take into consideration the current limitations of the guideline adaptation process. Evaluation of resources needed and the effect of the guideline adaptation for the success of the whole guideline implementation and practice change process is important for the development of future frameworks. By increasing the understanding and upfront estimation of resource (human and material) and time needed for the adaptation process, guideline implementers and adaptors will be able to decide which frameworks meet their needs.

The massive time and expertise requirements of some frameworks may make adaptation impractical in some contexts. Increasing the flexibility of adaptation frameworks can also help adaptors to modify the process to respond to different challenges that may arise in various guideline adaptation contexts. Training of the local guideline adaptation team before the adaptation process begins could also potentially minimize some of the difficulties with the expertise required for the utilization of the frameworks. As the frameworks are evolving, the impact of the modifications made to the frameworks to expedite the process needs to be further evaluated to ascertain the validity of the resultant recommendations and the advantages in terms of resources expended.

Although different implementation strategies may also be required for different contexts, most frameworks address only the adaptation process. An exception is the CAN-IMPLEMENT framework, which includes detailed steps for implementation, evaluation, and sustainability assessments for the adapted guidelines. Parts of this framework could be included in future frameworks. The presentation and dissemination of adapted guidelines is vital to their uptake; packaging the recommendations with a separate implementation manual and practice/behavior change interventions could be explored.

The frameworks are all presented from the perspectives of the local level guideline adapters or framework developers and focused on their own processes. From the perspective of guideline developers such as WHO or NHMRC, publishing a guideline that is adaptable could be critically important in assisting the local adaptation process.

The source guideline developers could potentially include a system for adaptation based on adaptation frameworks into their “implementation recommendations” section of future guidelines. This section could include an estimation of the time and resources (human and fiscal) need for adaptation, as well as the advantages and limitations of different adaptation frameworks. It could also describe which recommendations in the guideline are open to some adjustments to suit the local context, with evidence tables to explain how far the adaptors can modify them (for example, for type 2 diabetes, the recommended initial treatment maybe metformin, but the guideline could also include the classes of drugs for diabetes and drug combination regimens that could potentially be substituted for it and specify which ones not to use). This will greatly increase the efficiency of the adaptation process as the end-users of the guideline will have a better idea for how far the adaptations can go.

The GRADE EtD tables may also be useful for this purpose by providing specific contextual information that the adaptor can compare and apply to their setting, adding local information for discussion. GRADE-ADOLOPMENT hinted at the need for a more widespread use of EtD tables to expedite their framework and facilitate decision making by the adaptors (i.e., whether to adopt, adapt, or de novo create recommendations) [19]. Including EtD tables for each recommendation in an international or national guideline would mean that the issues and evidence that underpin the global or regional recommendation are explicit (e.g., balance of benefits and harms, acceptability of the intervention, burden of disease, resource availability). Local adapters can then update the EtD tables with local considerations and data, leading to locally relevant and acceptable recommendations, whether adopted or adapted. It remains to be determined how flexible such considerations should be at the local level as recommendations must stay true to the evidence on the balance of benefits and harms and other considerations in order to be valid.

Currently, no single adaptation framework can be used for all guidelines or all contexts. In addition to choosing to follow a framework that suits the setting of guideline adaptation, local guideline developers must also focus on capacity building in adaptation methods and collaboration with the local stakeholders to implement optimal guidelines for the local context. Capacity building in adaptation methods could help achieve the full potential of the frameworks. This could potentially be done by collaboration between major international guideline developers and local stakeholders, and training of local guideline developers and policy-makers in the methods of adaptation frameworks. With better knowledge in adaptation methods, the local adaptors can expedite the process of adaptation [32].

## Conclusion

We compared adaptation frameworks that are currently available in the literature. Advantages and limitations of these frameworks were identified. The main advantages of frameworks include the following: first, the methodological rigor of the process that leads to evidence-based adapted guidelines. With the evolution of the framework from adapting from a range of source guidelines, to adapting recommendations from within a single guideline, to constructing evidence tables for each recommendation, the frameworks are becoming more evidence focused. Second, the clearly laid out steps of adaptation frameworks provide structure to the process and increases the transparency for future groups to understand, evaluate, and/or imitate the process.

Some limitations of the frameworks were also identified. First, most adaptation frameworks have been developed and utilized in high-income settings. Second, many frameworks lack formal evaluation of their impact on the ultimate uptake of the adapted guidelines and patient outcomes. Third, many of the frameworks are resource and time consuming. Fourth, the frameworks often do not describe how to implement the adapted guideline.

We argue that the utilization of frameworks in the guideline implementation process can be optimized by:

1. Increasing the understanding and upfront estimation of resource and time needed and flexibility of adaptation frameworks to respond to different challenges that arise in various guideline adaptation contexts.
2. Capacity building in adaptation methods (i.e., collaboration with local stake holders in development and implementation of adaptation methods and adapted guidelines). A collaboration between international guideline developers (e.g., WHO) and local stakeholders could provide methodological expertise and take local needs into account.
3. Increasing the adaptability of the source recommendation document (e.g., WHO or NHMRC guidelines). The developers could potentially include a system for adaptation based on adaptation frameworks into their implementation recommendations section of future guidelines.
4. Adaptation frameworks should be rigorously tested to assess the usability of the frameworks as well as to evaluate the effectiveness of the frameworks in improving guideline implementation and uptake in different settings. Adaptation is a key step in the implementation process of guidelines, especially in the implementation of international guideline in a variety of contexts. The refinement of the current adaptation frameworks and the process of guideline adaptation would be an important step forward in changing health behaviour (of clinicians and general population alike) and the grand quest of improving global health. The idea of increasing the adaptability of guidelines has been a recent focus of WHO [35]. The effect of integrating adaptation methods such as optimized adaptation frameworks into the implementation sections of source recommendation documents (e.g., WHO guidelines) would be an important area to explore in future studies.

## Additional file

Additional file 1: **Table S1.** Factors influencing local guideline group’s decisions about how to construct recommendations for a new guideline. Table S2. Steps for the implementation of guidelines. (DOCX 31 kb)

## Abbreviations

AAP: The Alberta Ambassador Program
AGREE: Appraisal of Guidelines for Research and Evaluation
CPG: Clinical practice guidelines
EtD: Evidence to decision (mainly referring to EtD tables)
GRADE: Grading of Recommendations Assessment, Development and Evaluation
MAGIC: Making GRADE the irresistible choice
NHMRC: Australian National Health and Medical Research Council
NIH: US National Institutes of Health
PGEAC: Practice guidelines evaluation and adaptation cycle
SGR: Systematic guideline review
WHO: World Health Organisation
