# Supplementary material for: The advantages and limitations of guideline adaptation frameworks
Source: Implement Sci. 2018 May 29;13:72. doi: 10.1186/s13012-018-0763-4 (PMC5975671; doi:10.1186/s13012-018-0763-4)
Supplement: Supplementary file 1 — Table S1. Factors influencing local guideline group’s decisions about how to construct recommendations for a new guideline. Table S2. Steps for the implementation of guidelines. (DOCX 31 kb) [file 13012_2018_763_MOESM1_ESM.docx]

Guideline adaptation takes local resources into account and modifies the international guidelines according to local needs. The magnitude of the difference between the source guideline development context and the adaptation/implementation context is evaluated by the adaptors. When the differences in resources (both human and fiscal) between the contexts are minute, it may be possible for recommendations to be adopted with few modifications .

**Supplementary file Table 1. Factors influencing local guideline group’s decisions about how to construct recommendations for a new guideline**

| **Factors** | **Example** |
| --- | --- |
| The differences in disease prevalence, thus affecting which recommendations from source guidelines are given priority. | Malaria may not be a prevalent issue in Canada (1), so the related recommendations in the source guideline may not be included in the adapted guideline. |
| The credibility and quality of the source guideline, with only the highest quality guidelines being considered for adaptation. | The Appraisal of Guidelines for Research & Evaluation (AGREE) Instrument (2) may be used to appraise and select guidelines for consideration by the local guideline committee. |
| The quality /certainty/ confidence in the evidence on which the recommendations in the source guideline are based | If a structured process, such as GRADE or rankings of levels of evidence, is used to assess the quality of the evidence underpinning each recommendation in a guideline, the local committee may select only recommendations based on high quality evidence |
| The currency of the evidence on which the recommendations in the source guideline are based | If the evidence is out-dated, the local guideline committee may choose to update the systematic reviews used to develop the recommendations in the source guideline and revise the recommendations as needed for the adapted guideline. |
| The strength of the recommendations in the source guideline. | If a structured process such as GRADE is used to rate the strength of recommendations, the local committee may choose to adopt or adapt only strong recommendations (even if they are based on low quality evidence). |
| The differences in the local healthcare context compared to the source guidelines’ context. | Certain recommendations may be beyond the capacity of the local health care system, so the local guideline committee may choose to ignore these recommendations or adapt them in ways that make them implementable in the local context. |
| The differences in values and preferences of the local population. | The importance of a certain health issue is to the population, the acceptability of the recommended intervention, or the importance of the outcomes impacted by a recommendation could influence the local committee to adopt or adapt certain recommendations. |

1. McCarthy AE, Morgan C, Prematunge C, Geduld J. Severe malaria in Canada, 2001–2013. Malar J. 2015;14:151.

2. Brouwers MC, Kho ME, Browman GP, Burgers JS, Cluzeau F, Feder G, et al. AGREE II: advancing guideline development, reporting and evaluation in health care. Cmaj. 2010;182(18):E839-42.

**Supplementary file Table 2. Steps for the implementation of guidelines**

| 1) Consult stakeholders to gain insights into the values and preferences of the local population, |
| --- |
| 2) **Adapt the guideline** to suit the local situation (e.g. disease prevalence, resource and capabilities of the local health system) |
| 3) Disseminate the adapted guideline through a variety of mediums (paper copy, digital publication, summary handbooks) |
| 4) Use active implementation techniques instead of passive dissemination. |
| 5) Monitor for the effectiveness of the implementation process and health outcomes. |

Search strategy

**Search terms**

**Medline**

| **#** | **Search Statement** | **Results** | **Annotation** |
| --- | --- | --- | --- |
| 1 | (guideline* and (adopt* or adapt* or contextuali?e)).tw. | 11376 |  |
| 2 | (guidance* and (adopt* or adapt* or contextuali?e)).tw. | 3583 |  |
| 3 | 1 or 2 | 14492 |  |
| 4 | (national adj3 (guideline? or guidance)).tw. | 9098 |  |
| 5 | (regional adj3 (guideline? or guidance)).tw. | 380 |  |
| 6 | (society adj3 (guideline? or guidance)).tw. | 2632 |  |
| 7 | (institute adj3 (guideline? or guidance)).tw. | 719 |  |
| 8 | (board adj5 (guideline? or guidance)).tw. | 307 |  |
| 9 | (ministry adj3 (guideline? or guidance)).tw. | 116 |  |
| 10 | (department adj3 (guideline? or guidance)).tw. | 382 |  |
| 11 | 4 or 5 or 6 or 7 or 8 or 9 or 10 | 13389 |  |
| 12 | (adopt* or adapt* or contextuali?e).tw. | 510948 |  |
| 13 | 11 and 12 | 831 |  |
| 14 | (technique? or strateg* or tool? or framework?).tw. | 2204652 |  |
| 15 | (efficacy or efficien* or effective* or competenc* or evaluat*).tw. | 4219501 |  |
| 16 | 3 or 13 | 14492 |  |
| 17 | 14 and 15 and 16 | 2833 |  |
| 18 | limit 17 to (english language and humans and (clinical trial, all or controlled clinical trial or journal article or meta analysis or multicenter study or observational study or randomized controlled trial or "review" or systematic reviews) and last 15 years) | 2049 |  |

**Embase**

Embase <1974 to 2017 March 14>

| **#** | **Search Statement** | **Results** | **Annotation** |
| --- | --- | --- | --- |
| 1 | (guideline* and (adopt* or adapt* or contextuali?e)).tw. | 20341 |  |
| 2 | (guidance* and (adopt* or adapt* or contextuali?e)).tw. | 6306 |  |
| 3 | 1 or 2 | 25673 |  |
| 4 | (national adj3 (guideline? or guidance)).tw. | 17086 |  |
| 5 | (regional adj3 (guideline? or guidance)).tw. | 815 |  |
| 6 | (society adj3 (guideline? or guidance)).tw. | 5326 |  |
| 7 | (institute adj3 (guideline? or guidance)).tw. | 1366 |  |
| 8 | (board adj5 (guideline? or guidance)).tw. | 508 |  |
| 9 | (ministry adj3 (guideline? or guidance)).tw. | 193 |  |
| 10 | (department adj3 (guideline? or guidance)).tw. | 674 |  |
| 11 | 4 or 5 or 6 or 7 or 8 or 9 or 10 | 25448 |  |
| 12 | (adopt* or adapt* or contextuali?e).tw. | 714063 |  |
| 13 | 11 and 12 | 1583 |  |
| 14 | (technique? or strateg* or tool? or framework?).tw. | 3187463 |  |
| 15 | (efficacy or efficien* or effective* or competenc* or evaluat*).tw. | 6302397 |  |
| 16 | 3 or 13 | 25673 |  |
| 17 | 14 and 15 and 16 | 5311 |  |
| 18 | limit 17 to (human and english language and (clinical trial or randomized controlled trial or controlled clinical trial or multicenter study) and (article or journal or "review") and last 15 years) | 405 |  |

**CINAHL**

| **#** | **Query** | **Limiters/Expanders** | **Last Run Via** | **Results** |
| --- | --- | --- | --- | --- |
| S18 | S14 NOT S17 | Search modes - Boolean/Phrase | Interface - EBSCOhost Research Databases  Search Screen - Advanced Search  Database - CINAHL | 495 |
| S17 | S15 OR S16 | Search modes - Boolean/Phrase | Interface - EBSCOhost Research Databases  Search Screen - Advanced Search  Database - CINAHL | 41,643 |
| S16 | (MH “Animals+”) NOT (MH “Human”) | Limiters - English Language; Published Date: 20020101-20171231; Publication Type: Clinical Trial, Journal Article, Meta Analysis, Randomized Controlled Trial, Research, Review, Systematic Review  Search modes - Boolean/Phrase | Interface - EBSCOhost Research Databases  Search Screen - Advanced Search  Database - CINAHL | 25,952 |
| S15 | TI ( rat or rats or cow or cows or chicken? or horse or horses or mice or mouse or bovine or animal? ) OR MW animal? | Limiters - English Language; Published Date: 20020101-20171231; Publication Type: Clinical Trial, Journal Article, Meta Analysis, Randomized Controlled Trial, Research, Review, Systematic Review  Search modes - Boolean/Phrase | Interface - EBSCOhost Research Databases  Search Screen - Advanced Search  Database - CINAHL | 40,635 |
| S14 | S9 AND S10 AND S11 | Limiters - English Language; Published Date: 20030101-20171231; Publication Type: Clinical Trial, Journal Article, Meta Analysis, Randomized Controlled Trial, Research, Review, Systematic Review  Search modes - Boolean/Phrase | Interface - EBSCOhost Research Databases  Search Screen - Advanced Search  Database - CINAHL | 496 |
| S13 | S9 AND S10 AND S11 | Limiters - English Language  Search modes - Boolean/Phrase | Interface - EBSCOhost Research Databases  Search Screen - Advanced Search  Database - CINAHL | 602 |
| S12 | S9 AND S10 AND S11 | Search modes - Boolean/Phrase | Interface - EBSCOhost Research Databases  Search Screen - Advanced Search  Database - CINAHL | 632 |
| S11 | TI ( efficacy or efficien* or effective* or competenc* or evaluat* ) OR AB ( efficacy or efficien* or effective* or competenc* or evaluat* ) OR MW ( efficacy or efficien* or effective* or competenc* or evaluat* ) | Search modes - Boolean/Phrase | Interface - EBSCOhost Research Databases  Search Screen - Advanced Search  Database - CINAHL | 708,897 |
| S10 | TI ( technique? or strateg* or tool? or framework? ) OR AB ( technique? or strateg* or tool? or framework? ) OR MW ( technique? or strateg* or tool? or framework? ) | Search modes - Boolean/Phrase | Interface - EBSCOhost Research Databases  Search Screen - Advanced Search  Database - CINAHL | 294,718 |
| S9 | S3 OR S8 | Search modes - Boolean/Phrase | Interface - EBSCOhost Research Databases  Search Screen - Advanced Search  Database - CINAHL | 3,384 |
| S8 | S6 AND S7 | Search modes - Boolean/Phrase | Interface - EBSCOhost Research Databases  Search Screen - Advanced Search  Database - CINAHL | 26 |
| S7 | TI ( adopt* or adapt* or contextuali?e) OR AB ( adopt* or adapt* or contextuali?e) OR MW ( adopt* or adapt* or contextuali?e) | Search modes - Boolean/Phrase | Interface - EBSCOhost Research Databases  Search Screen - Advanced Search  Database - CINAHL | 74,823 |
| S6 | S4 OR S5 | Search modes - Boolean/Phrase | Interface - EBSCOhost Research Databases  Search Screen - Advanced Search  Database - CINAHL | 461 |
| S5 | AB ( (national n3 (guideline*1 or guidance )) ) OR AB ( (regional n3 (guideline*1 or guidance )) ) OR AB ( (society n3 (guideline*1 or guidance )) ) OR AB ( (association n3 (guideline*1 or guidance )) ) OR AB ( (institute n3 (guideline*1 or guidance )) ) OR AB ( (ministry n3 (guideline*1 or guidance )) ) OR AB ( (department? n5 (guideline*1 or guidance )) ) | Search modes - Boolean/Phrase | Interface - EBSCOhost Research Databases  Search Screen - Advanced Search  Database - CINAHL | 367 |
| S4 | TI ( (national n3 (guideline*1 or guidance )) ) OR TI ( (regional n3 (guideline*1 or guidance )) ) OR TI ( (society n3 (guideline*1 or guidance )) ) OR TI ( (association n3 (guideline*1 or guidance )) ) OR TI ( (institute n3 (guideline*1 or guidance )) ) OR TI ( (ministry n3 (guideline*1 or guidance )) ) OR TI ( (department? n5 (guideline*1 or guidance )) ) | Search modes - Boolean/Phrase | Interface - EBSCOhost Research Databases  Search Screen - Advanced Search  Database - CINAHL | 103 |
| S3 | S1 OR S2 | Search modes - Boolean/Phrase | Interface - EBSCOhost Research Databases  Search Screen - Advanced Search  Database - CINAHL | 3,383 |
| S2 | AB ( (guideline* and (adopt* or adapt* or contextuali?e)) OR AB ( (guidance and (adopt* or adapt* or contextuali?e) ) | Search modes - Boolean/Phrase | Interface - EBSCOhost Research Databases  Search Screen - Advanced Search  Database - CINAHL | 3,240 |
| S1 | TI ( (guideline* and (adopt* or adapt* or contextuali?e)) OR TI ( (guidance and (adopt* or adapt* or contextuali?e) ) | Search modes - Boolean/Phrase | Interface - EBSCOhost Research Databases  Search Screen - Advanced Search  Database - CINAHL | 200 |
